# Evaluation of an Assertive Continuing Care Program for Hispanic Adolescents

**DOI:** 10.5539/gjhs.v7n5p106

**Published:** 2015-02-24

**Authors:** Eric Strunz, Joanna Jungerman, Juliet Kinyua, Paula M. Frew

**Affiliations:** 1 Department of Behavioral Science and Health Education, Rollins School of Public Health, Emory University, Atlanta, Georgia, USA; 2 Division of Infectious Diseases, Department of Medicine, Emory University School of Medicine, Atlanta, Georgia, USA

**Keywords:** Hispanic, adolescents, substance abuse, program evaluation, zero inflation, correlated data

## Abstract

**Purpose:**

This study evaluated an Adolescent Community Reinforcement Approach (A-CRA) and Assertive Continuing Care (ACC) program targeting Hispanic adolescents at risk for substance abuse.

**Method:**

The Clinic for Education, Treatment, and Prevention of Addiction (CETPA, Inc.), a behavioral health provider offering culturally appropriate substance use and mental health services, carried out the intervention. We examined longitudinal substance use data in relation to time spent in the program and possible confounders.

**Results:**

We analyzed data from 72 adolescent clients collected between 2010 and 2012. Self-reported data were evaluated to determine if time spent in the program was associated with substance use reduction. The data were correlated, zero-inflated, and overdispersed; consequently, we employed a mixed-effects zero-inflated negative-binomial model. Time spent in CETPA’s program was significantly associated with reductions in the number of days of substance use (p = .039), but not with the likelihood of fully abstaining from use (p = .290). For non-abstinent participants who spend a year in the program, our models revealed an average decline of 46% in reported days of substance use.

**Conclusions:**

A culturally tailored and age-appropriate substance abuse program for Hispanic adolescents resulted in a significant reduction of the numbers of days using alcohol, drugs, or other illicit substances. The A-CRA/ACC approach can yield successful results in culturally diverse settings.

## 1. Introduction

Substance abuse remains a significant problem for adolescents. Some 37% of US adolescents aged 12 to 17 years reported using alcohol or illicit drugs in the previous year, and 7.9% of those met the criteria for a substance abuse disorder (Wu, Woody, Yang, Pan, & Blazer, 2011). Adolescents who drink or use marijuana regularly show inferior cognitive skills and are more likely to exhibit delinquent behaviors (Komro et al., 1999; Lisdahl, Gilbart, Wright, & Shollenbarger, 2013). Marijuana use may also lead to lower educational attainment, higher rates of school dropout, and other illicit drug use (Lynskey & Hall, 2000; Macleod et al., 2004). Substance use may also have a broader societal impact, as alcohol and illicit drug use are linked to 23% of the global burden of disease in developed countries (Toumbourou et al., 2007).

Due to the high risk of substance use initiation in adolescence, teenage populations are a key target of prevention and intervention efforts. Healthy People 2020 lists several objectives concerning adolescent substance use, including targets for reductions in past-month use of alcohol, marijuana, and other illicit drugs among youth (ages 12-17) (U.S. Department of Health and Human Services - Office of Disease Prevention and Health Promotion, 2010). While reductions in most of these areas have been achieved, marijuana use increased between 2008 and 2011 (Substance Abuse and Mental Health Services Administration [SAMHSA], 2012).

Latino youth have a high prevalence of both substance use (21%) and disorders (7.7%) (Wu et al., 2011). Obtaining inpatient care and outpatient treatment for these problems can be challenging as nearly a third of the Latino population lacks health insurance. Language barriers contribute to additional disparities in healthcare access (DeNavas-Walt, Proctor, & Smith, 2011; Kim et al., 2011). Racial and ethnic minority populations are less likely than non-minority counterparts to receive needed mental health services. They also are more likely to receive poor-quality care (Office of the Surgeon General, Center for Mental Health Services, & National Institute of Mental Health, 2001). This elevated risk is especially significant considering that Latinos are both the fastest growing and largest minority group in the U.S., comprising 15.8% of the population in 2009 (DeNavas-Walt et al., 2011).

Numerous strategies have been developed to help curb adolescent substance use, ranging from student assistance programs in schools to full residential treatment. Continuing care strategies have become increasingly integrated into substance use treatment, especially for adolescents. The Adolescent Community Reinforcement Approach (A-CRA) and Assertive Continuing Care (ACC) are two related interventions that have yielded success in reducing adolescent substance use (Meyers, Roozen, & Smith, 2011; Roozen et al., 2004). ACC is an approach that shifts the responsibility for engagement in care from the patient to the clinician, thereby increasing the likelihood that patients remain in treatment (Godley, Godley, Dennis, Funk, & Passetti, 2007). For example, ACC programs provide “assertive” services, such as individual case management, home visits, and family counseling. ACC was devised to enhance continuing care services after post-residential treatment.

A-CRA is an offshoot of the Community Reinforcement Approach (CRA), a successful adult substance abuse treatment program. A-CRA is specifically tailored to meet the unique needs of adolescents. The goal of both programs is to help patients create a drug-free lifestyle that is more rewarding and enjoyable than one involving substance use (Meyers et al., 2011). Through skill-building workshops, counseling, and engagement in drug-free social activities, adolescents learn to better avoid situations that are conducive to substance use (Godley et al., 2002). ACC and A-CRA strategies are often used in combination to further increase compliance with program activities (Godley & Godley, 2010). In April 2014, over 150 agencies across the country were implementing or beginning to implement A-CRA/CRA/ACC services (Chestnut Health Systems, 2015).

## 2. Materials & Method

### 2.1 Material Studied

Previous evaluations of the A-CRA/ACC approach have resulted in favorable findings (Meyers et al., 2011). A randomized trial found that adolescents assigned to the ACC treatment decreased their percentage of days using alcohol significantly more than those assigned to usual care (64% vs. 18% reduction) (Godley et al., 2002). The study also found that more ACC participants reported abstinence from substance use compared to those receiving usual care at nine-month follow up (41% vs. 26%). Another investigation found that 68.7% of adolescents reporting ceasing cannabis use within 12 months (McGarvey & Leon-Verdin, 2012). These past studies suggest that the A-CRA/ACC approach can reduce quantity of substance use as well as increase rates of abstinence in adolescent populations.

Although the A-CRA/ACC approach has been previously validated, it is less clear that the approach is effective in racially and culturally diverse settings. Cultural context represents a critical consideration for program effectiveness, since A-CRA requires family involvement, home visits, and lifestyle re-organization. In order to strengthen the evidence exploring A-CRA/ACC in diverse populations, this study examines a behavioral health provider which targets a predominantly Hispanic group of adolescents.

### 2.2 Area Descriptions

The Clinic for Education, Treatment, and Prevention of Addiction (CETPA, Inc.) is a behavioral health provider that offers culturally appropriate services. Founded in 1999, CETPA is the only licensed agency in the state of Georgia that provides behavioral health treatment and prevention services in English and Spanish. Employing a team of bilingual and bicultural clinicians, CETPA operates an A-CRA/ACC program as part of an initiative sponsored by the Substance Abuse Mental Health Services Administration (SAMHSA). This federal initiative also includes 33 other sites across the United States. As part of the grant reporting process, CETPA periodically collects data from participants about substance use, risk factors, and other valuable indicators. This information forms a longitudinal data set that can be analyzed to assess programmatic outcomes.

Therapists at CETPA provide each adolescent client with 12 office sessions, 12 in-home sessions and two family-client joint sessions over the course of about six months. Sessions usually last one hour but can vary according to the therapist’s discretion. If needed, therapist transport clients to and from appointments and administer urine drug tests. The length of time spent in the pro-gram varies based on the needs and progress of each individual client.

In addition to the standard six-month A-CRA/ACC program, clients at CETPA are typically enrolled in a clubhouse program that is available to participants during all 12 months of follow-up. The clubhouse provides adolescents with a supportive after-school environment, including home-work assistance, games, exercise equipment, and group meals. Participants build stronger relation-ships with staff members and each other, potentially replacing negative peer relationships with positive ones. The clubhouse also offers a supplemental support group for parents of adolescents enrolled in A-CRA.

The purpose of this study was to evaluate if time spent in CETPA’s A-CRA/ACC program resulted in reduction of self-reported substance use. We hypothesized that completing the program at 12 months would be associated with approximately 50% reduction in number of days of reported substance use. Previous research suggests that the time spent in a program serves as a valuable indicator of participant adherence and success (Godley, Godley, Dennis, Funk, & Passetti, 2007), so additional investigation on the temporal relationship between program completion and substance use is warranted. Godley and colleagues (2007) investigated the effect of assertive continuing care (ACC) versus usual continuing care (UCC) on linkage to care, retention and adherence to treatment in adolescents post-residential treatment. The authors found that ACC was significantly better than UCC at retaining patients in care and supporting long term abstinence from marijuana. The present study differs from the aforementioned work in two major ways. First, all patients enrolled in A-CRA/ACC at CETPA have a diagnosis of drug or alcohol dependence, but they have not necessarily been treated in a residential setting. Second, 90% of enrolled subjects in the present study identified as Hispanic, while only 3% identified as such in the Godley study.

### 2.3 Methods

Persons eligible for inclusion in this study were systematically referred to the A-CRA/ACC program offered at CETPA via the juvenile justice system, schools, or their families. The participants selected to enroll in the program included 72 adolescent clients aged 13 to 18 years. Eligibility was limited to those who had a Diagnostic and Statistical Manual of Mental Disorders IV (DSM IV) diagnosis of alcohol or drug dependence. A structured clinical interview for DSM-IV was used for diagnosis.

Data were collected via the Global Appraisal of Individual Needs (GAIN) self-report survey instrument (Dennis, Titus, White, & Unsicker, 2008). These questionnaires were administered at four time points within 12 months: entry into the program (baseline), 3 months, 6 months, and 12 months. Data were collected between November 2010 and September 2012. Participants were asked to report the number of days that they used various substances in the past 30 days. Our primary outcome of interest was “any substance use,” though substances other than alcohol and marijuana were rare. The data set included an array of outcome measures and demographic details; Table 1 provides an overview of these variable groups with specific examples from each. The data were unbalanced, since participants entered the program at different times, yet they were also time-structured, since all participants were surveyed using the same measurement schedule (i.e., baseline, 3, 6, 12 months). Consequently, we analyzed an anonymized time-series data set containing 230 measurement occasions from the 72 participants.

**Table 1:** Longitudinal measurement variables

Variable Category	Specific Example(s)
Intake / Admin	Date when client began receiving services
Recommended Services	What services were recommended at intake?
Demographics	Age, ethnicity
Personal Details	Living arrangements, client parental status
Substance Abuse	Self-reported marijuana use in past 30 days
Education & Wages	Highest educational attainment, monthly income
Criminal Justice	Number of days spent under arrest in past month
Health Services Used	Any hospitalizations
Mental Health	Experiences with depression, anxiety
Social Connectedness	Attendance of religious services, group gatherings
Sexual Activity	Unprotected sex occasions

**Figure 1:**
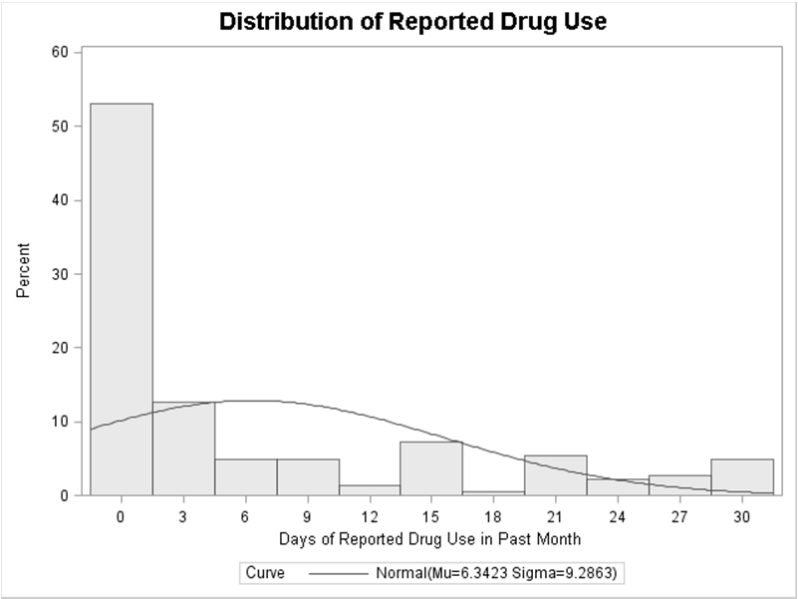
Distribution of self-reported days of drug use in past month

We anticipated that data were correlated due to their longitudinal nature, so we employed random effects in our model. Traditional analytic approaches assume that all observations are statistically independent, and failing to account for correlated data can lead to inaccurate standard errors, biasing the statistical significance of estimates (Cannon, Warner, Taddei, & Kleinbaum, 2001). Strong non-normality was also present (Figure 1), which functional and geometric transformations failed to address, so we pursued a count-model approach. The data showed many zero outcomes (n=104), with almost half of all responses indicating no drug use in the past month. Overdispersion was also present, with the variance greatly exceeding the mean, even after adjusting for the excess zeroes. We thus adopted a two-part model to account for “zero inflation” and a negative binomial distribution to address overdispersion. Combined with the random effects needed for the longitudinal design, our analytic strategy consisted of a mixed-effects, zero-inflated, negative-binomial model (ZINB).

Zero-inflated models have been used successfully in many disciplines (Chipeta, Ngwira, & Kazembe, 2013; Yau, Wang, & Lee, 2003a), though care is needed in interpreting their results. Count distributions (e.g., Poisson, negative binomial) will typically produce some zero values. However, if there is a large excess of zeroes, then the supplemental zero values may be caused by a separate process (Lambert, 1992). For example, data could potentially come from two groups: individuals who are truly abstinent (excess zeroes) and others that do use substances periodically, but not necessarily every month. The zero-inflated approach analyzes these groups separately and combines them with an overarching, linked model. Different explanatory variables may be important for one group, but not the other. Results from a zero-inflated model therefore provide information about two topics: variables that are associated with a higher likelihood of being in the “zero group” and variables that are associated with higher count values. We also considered a hurdle model, which assumes that all zeroes are formed from a unique process (Mullahy, 1986). A zero-inflated approach seemed more reasonable however, since individuals at risk for substance abuse may not use drugs every month.

The probability distribution of a zero-inflated negative binomial random variable, Y, is given by:


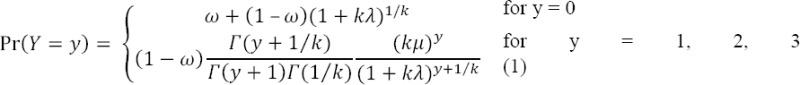


Where λ is the mean of the underlying negative binomial distribution, k is the negative binomial dispersion parameter, and ω is the probability of an observation being drawn from the separate distribution that always generates zeros (Ridout, Demétrio, & Hinde, 1998). The lowercase omega (ω) represents the zero-inflation probability (e.g., probability of being in the zero group). The uppercase gamma (Γ) refers to the gamma function.

To address the data’s longitudinal nature, we used a mixed effects modeling strategy. Mixed effects refer to models that use both “fixed” and “random” effects, where fixed effects denote estimated group averages (parameters) and random effects deal with subject-specific variations (Singer & Willett, 2003). By accounting for the natural diversity among clients, we more accurately assess what role important fixed effects, like time in program, have on the outcome (Cannon et al., 2001). We used a random-effect component for the model’s intercept—both on the zero and count models—which enabled the analysis to consider different baseline values for participants. The random effects for both components were assumed to be independent from one another and distributed as N (0,σ2u) and N(0,σ2v) respectively. Our model did not converge when a random slope was added, so the term was subsequently dropped.

To build our model, we conducted bivariate analysis to assess the crude relationship between each variable and the outcome (number of days of reported use of any substance). Time spent in program was treated as the independent variable (i.e., exposure). Time in the program was transformed to reflect three-month intervals, so the variable could take values of 0, 1, 2, or 4. We also assessed pre-specified variables and interactions that were deemed potentially relevant from the literature review and stakeholder consultations (e.g., ethnicity, sex). Variables which showed at least a weak association with the outcome (p-value <.2) were included for subsequent consideration. We then proceeded to manually fit the model using backwards elimination with the likelihood-ratio test. Our final model was:

- Zero Model: Intercept + Time in Program + Sex + Days Confined
- Count Model: Intercept + Time in Program

Analysis was performed with SAS 9.3 (Cary, NC) using the NLMIXED procedure. Our modeling syntax was informed by examples documented through the SAS-L listserv (McLerran, 2007) and other generalized linear mixed model (GLMM) applications (McGilchrist, 1994; Yau, Wang, & Lee, 2003b).

## 3. Results

Most of the 72 participants were male (72%) and many of them were ages 15 to 16 (61%) with a range from 11 to 18 years of age. The majority of the participants in the program reported having Hispanic ethnicity (90%, n=65) and Mexican race (71%, n=51). Other country/regional heritage reported included Central American (13%, n=9), South American (3%, n=2), Cuban (1%, n=1), Dominican (1%, n=1), and unknown (11%, n=8). Notably, the proportion of Hispanic and Latino adolescents in our sample was much higher than other samples cited in the literature, which ranged from 0% to 32% (Godley et al., 2002; M. Dennis et al., 2004; Slesnick, Prestopnik, Meyers, & Glassman, 2007; McGarvey & Leon-Verdin, 2012; Godley, Hedges, & Hunter, 2011).

Program follow-up revealed that most participants had completed intervention activities at six months, but fewer had completed the full twelve months. Three months into the program, 68 of the 72 (94%) adolescents completed their follow-up assessment. Six months into the program, 56 participants (78%) completed follow-up, while an additional 34 (47%) completed the survey at twelve months. Historically, CETPA has maintained ≥70% 12-month follow-up rate in its programs. The lower follow-up rate at 12 months in this sample reflects the fact that many participants (n=31) had not yet been enrolled in the study for a full year; this is a consequence of conducting an evaluation of an ongoing community program. Further, no indication was found of systematic causes of missingness in the data.

A numerical breakdown of the mean days of substance use over time, along with the frequency of any substance use, contextualizes this downward trend (Table 2). Little change in drug abstinence was observed over time, but there is a 34% reduction in average days of substance use between baseline and 12 months.

**Table 2:** Reported use of any substance in past month by time in program

Time	Number of Clients	Mean Days of Use	% Reduction from Baseline Average	Zero Use (Abstinence)
Baseline	72	7.51	N/A	34 (47%)
3 months	68	6.26	17%	31 (46%)
6 months	56	5.62	25%	25 (47%)
12 months	34	4.93	34%	14 (48%)

To ensure that our model was appropriate, we evaluated negative binomial and random effect components through likelihood ratio tests, with these tests yielding p-values≤0.02. While the random intercept component for the zero model may not appear significant from its standard error, likelihood-ratio tests confirmed the superiority of the model that included both random intercept effects (p=.003). We also included a random covariance term to assess the assumption of independence between random effects, and the covariance was found to be non-significant (p=.403). As a result, the reduction in average number of days of substance abuse from baseline to 12 months indicates an effect that is likely due to program participation.

Testing the zero-inflation strategy is more complex, since a one-part negative binomial model is not nested within a two-part ZINB model. However, the intercept value can help assess the need for zero inflation (Erdman, Jackson, & Sinko, 2008). A large negative constant for the zero intercept suggests that the zero-inflated segment is small. In other words, a significantly negative parameter estimate for the zero model intercept implies that a ZINB approach is equivalent, and therefore inferior, to a simpler negative binomial model. In our model’s estimates, the zero model’s intercept was positive (.630) and not significant (p=.227). We also compared Akaike’s Information Criterion (AIC) statistic (Akaike, 1998) between the final model (AIC=1102) and a basic negative-binomial random-effects model with time as the only predictor (AIC=1143). The final model’s lower AIC statistic suggests that it is preferable to the comparison model which did not include zero inflation.

Results from both the zero model, which included “sex” and “number of confined days” as covariates, and the count model, which included no additional covariates, provide useful insights. The zero model analyzed the odds of being in the “true zero” or abstinent group. These were individuals considered unlikely to report any drug use. The odds of females being abstinent were 1.878. In comparison to females, males showed 0.271 the odds (less than one third) of being abstinent, which was statistically significant (p=.029). Days spent in some type of confinement (e.g., incarceration, rehab) in the previous month also emerged as an important factor. For every additional day spent in confinement during the past month, the odds for being abstinent increased by a factor of 1.186, a significant association (p=.018). For example, a female who spent five days confined (e.g., incarcerated) would have over twice the odds (2.347) of being abstinent compared with a female who spent no time in confinement. However, time spent in the program did not have a significant association with abstinent behavior (p=.290). In other words, spending time in the A-CRA/ACC program did not appear to affect the chances of participants being abstinent.

The count model analyzed the expected number of days of substance use for participants that were not “true zeros” (i.e., non-abstinent). For adolescent clients at baseline, the expected number of days of substance use was about 10. If non-zero participants were to increase intervention time by three months, the expected number of days of substance use would decrease by a factor of 0.856, which was statistically significant (p=.039). For participants who spent all 12 months in the program, we would expect an average 46.3% decline in reported days of substance use. Additional parameter estimates are available in Table 3.

**Table 3:** Parameter estimates from ZINB model with random effects

Model	Parameter Name	Estimate	Std. Err.	P-value	Anti-log	95% CI
Count	Count intercept	2.3316	0.1770	<.0001	10.294	7.233, 14.651
Count	Time (3 months)	-0.1556	0.0741	0.0393	0.856	0.738, 0.992
Zero	Zero intercept	0.6304	0.5170	0.2268	1.878	0.67, 5.268
Zero	Time (3 months)	-0.1815	0.1702	0.2901	0.834	0.594, 1.171
Zero	Male	-1.3061	0.5867	0.0292	0.271	0.084, 0.873
Zero	Confined Days	0.1706	0.0703	0.0178	1.186	1.031, 1.365
Zero	Random Int. Zero	2.0256	1.2348	0.1054		
Count	Random Int. Count	0.6524	0.2685	0.0177		
Count	Overdispersion	0.4872	0.1454	0.0013		

## 4. Discussion

Time spent in the CETPA program was significantly associated with reduced substance use over time (p=.039), but not with the likelihood of completely abstaining from substance use (p=.290). For non-abstinent participants who spend all 12 months in the program, we would expect an average 46.3% decline in reported days of substance use. Even a three-month program involvement would be expected to reduce days of substance use by 14.4%. From these estimates, we would expect an average non-abstinent participant to use substances about 10 days a month at the beginning of the program, 8.6 days per month after 3 months, and 5.4 days after one year. These results are relatively similar to past trials despite differences in study population (Slesnick et al., 2007).

Although we did not find a statistically significant increase in adopting abstinent behavior, substance use reduction remains a more pressing outcome for current interventions. Harm reduction has emerged in recent years as a critical paradigm for interventions aimed at a variety of negative behaviors, supplanting previous perspectives that focused on abstinence alone (Beck, 1998; Canadian Paediatric Society, 2008; Toumbourou et al., 2007). Reducing use has proven to be particularly important for curbing the impact of alcohol among adolescents (Masterman & Kelly, 2003; McBride, Farringdon, Midford, Meuleners, & Phillips, 2004; Neighbors, Larimer, Lostutter, & Woods, 2006). While abstinence remains an ideal goal in some drug control paradigms, harm reduction appears to be a more realistic and largely equally valuable outcome.

Both recent confinement and sex emerged as important factors for exploring drug abstinence. Compared with females, males had less than one-third likelihood of being abstinent (OR=.271, p=.029). This relationship emerges logically from previous research which has found that males are at increased risk of substance use during adolescence and early adulthood (SAMHSA, 2012). Days spent in some type of confinement (e.g., incarceration, detention) in the previous month were also included in the zero model. For every additional day spent in confinement during the past month, the odds of being abstinent increased by a factor of 1.186 (p=.018). For example, a female who spent five days in jail would have over twice the odds (2.347) of being abstinent compared with a female who spent no time in confinement. This relationship seems plausible, since adolescents in confinement would likely have reduced access to substances.

This study advances our understanding of the structure and timing of substance use programs that are effective for Hispanic adolescents. Previous studies have broadly validated the effectiveness of A-CRA/ACC, but our sample is unique in terms of ethnic and cultural characteristics. Ninety percent of participants in this study self-identified as Hispanic, whereas previous research has examined groups comprising of between 0% and 32% Hispanic individuals (Godley et al., 2002; Dennis et al., 2004; Slesnick et al., 2007; McGarvey & Leon-Verdin, 2012; Godley et al., 2011).

Our results provide evidence that A-CRA/ACC can be successfully implemented with Hispanic adolescents. Factors such as country of heritage or origin, cultural identity, and preference for Spanish as a primary language are important considerations for structuring program activities. In particular, replication of A-CRA/ACC interventions in Hispanic populations remains critical due to the important role that culture may have in the treatment process, especially for psychotherapeutic interventions (Bernal, Bonilla, & Bellido, 1995; Bernal & Scharrón-del-Río, 2001; Tharp, 1991). Previous studies have noted the scarcity of information that validated evidence-based treatments for ethnic minorities in real-world, non-academic settings (Godley et al., 2011). Our study helps expand knowledge in that domain, evaluating a program conducted by a community treatment organization—not a university clinic—that also serves predominantly Hispanic clientele.

This evaluation also provides context for future investigations into the impact of culture on the structure and outcome of substance abuse treatment programs (e.g., close participant monitoring, availability of ongoing social activities for participants, and behavioral monitoring of substance use at quarterly time points). Differences in acculturation, immigration status, language proficiency, and other cultural characteristics are likely to significantly influence program accessibility, retention, and success. In-depth qualitative research may be needed to further understand how A-CRA/ACC can be best implemented across varying ethnic and cultural contexts.

For counseling practitioners, these results validate the value of the A-CRA/ACC approach. Our findings replicate previous randomized trials within the “real-world” context of a community health clinic serving a Hispanic audience. This successful replication further reinforces the value of using evidence-based practices.

For researchers and evaluators, our analytic method represents a flexible approach that could empower future intervention assessments. Mixed-effects zero-inflated models do not require that data be normally distributed and can also adjust for longitudinal study designs. Previous evaluations of A-CRA/ACC programs have used generalized estimating equations (GEE) (McGarvey & Leon-Verdin, 2012) and the f-index (Godley et al., 2002) to analyze outcome data. A zero-inflated model with random effects provides a potentially more flexible analytic strategy that utilizes all available data, especially when normality of data cannot be established. The model may be difficult to fit, however, especially if data are limited. Previous analyses have also predominantly used SPSS, which is not natively capable of fitting a mixed zero-inflated model (IBM, 2013).

The use of a zero-inflated model also requires caution when interpreting results, since the analysis assumes two processes are occurring: one yields values with a count distribution (the “count model”) and another produces excess zeroes (the “zero model”). The analysis does not investigate, however, what phenomenon is actually causing the extra zeroes. After consulting the literature and subject matter experts, we have assumed that the zero process is linked to drug abstinence. In other words, our sample is theorized to be comprised of those who are abstinent from drugs and those who are not. An alternative explanation, though not supported by any firm evidence, might be that excess zeroes are caused by individuals who purposefully underreport their substance use. In that scenario, the zero model would analyze the likelihood of reporting behavior honestly or not. Associating the zero model with drug abstinence seems like a logical connection—and a helpful interpretation tool for others—but readers are encouraged to approach this detail with care.

This analysis did not include a control group or experimental design, so we cannot establish a direct causal relationship between the A-CRA/ACC program and reduced substance use. However, participants did experience a similar reduction in substance use compared to past-randomized controlled trials. The progression of time is an important potential confounder, since aging or other environmental changes may influence behavior. Prior research suggests that substance use typically rises during teenage years, peaking in the mid-20’s. Considering that most of our participants were 15 or 16 at program entry, it seems unlikely that their reduced substance use was caused by maturation alone. However, other concurrent effects, like family intervention efforts, cannot be discounted.

A majority of CETPA’s adolescent clients participated not just in the A-CRA/ACC intervention, but also a complementary clubhouse program. Analyzing data from individuals that participated in this added service provides valuable perspective into the working reality of community clinics, but also creates additional limitations for inferring a causal connection between the A-CRA/ACC program and reduced substance use. Available data did not allow us to distinguish between individuals who were or were not participating in the clubhouse. Future research could more directly assess the potential synergy of parallel services on A-CRA/ACC participant outcomes.

## 5. Conclusion

Participants in CETPA’s A-CRA/ACC program showed a significant decline in number of days of substance use, though a significant improvement in the initiation of abstinent behavior was not observed. Approximately 90% of our study sample was Hispanic, and our results add confirmation to past research findings that this approach is useful for substance abuse reduction across cultural boundaries.
